# *Aedes Albopictus* and Cache Valley virus: a new threat for virus transmission in New York State

**DOI:** 10.1080/22221751.2022.2044733

**Published:** 2022-03-03

**Authors:** Constentin Dieme, Joseph G. Maffei, Maryam Diarra, Cheri A. Koetzner, Lili Kuo, Kiet A. Ngo, Alan P. Dupuis II, Steven D. Zink, P. Bryon Backenson, Laura D. Kramer, Alexander T. Ciota

**Affiliations:** a New York State Department of Health, Wadsworth Center, Slingerlands, NY, USA; b Institut Pasteur de Dakar, Dakar, Senegal; c New York State Department of Health, Bureau of Communicable Disease Control, Albany, NY, USA; d Department of Biomedical Sciences, School of Public Health, State University of New York at Albany, Albany, NY, USA

**Keywords:** Cache Valley virus, *Aedes albopictus*, New York State, mosquitoes, surveillance, vector competence, vector-borne infections, viruses

## Abstract

We report surveillance results of Cache Valley virus (CVV; *Peribunyaviridae*, *Orthobunyavirus*) from 2017 to 2020 in New York State (NYS). Infection rates were calculated using the maximum likelihood estimation (MLE) method by year, region, and mosquito species. The highest infection rates were identified among *Anopheles* spp. mosquitoes and we detected the virus in *Aedes albopictus* for the first time in NYS. Based on our previous *Anopheles quadrimaculatus* vector competence results for nine CVV strains, we selected among them three stains for further characterization. These include two CVV reassortants (PA and 15041084) and one CVV lineage 2 strain (Hu-2011). We analyzed full genomes, compared *in vitro* growth kinetics and assessed vector competence of *Aedes albopictus*. Sequence analysis of the two reassortant strains (PA and 15041084) revealed 0.3%, 0.4%, and 0.3% divergence; and 1, 10, and 6 amino acid differences for the S, M, and L segments, respectively. We additionally found that the PA strain was attenuated in vertebrate (Vero) and mosquito (C6/36) cell culture. Furthemore, *Ae. albopictus* mosquitoes are competent vectors for CVV Hu-2011 (16.7–62.1% transmission rates) and CVV 15041084 (27.3–48.0% transmission rates), but not for the human reassortant (PA) isolate, which did not disseminate from the mosquito midgut. Together, our results demonstrate significant phenotypic variability among strains and highlight the capacity for *Ae. albopictus* to act as a vector of CVV.

## Introduction

Cache Valley virus (CVV; *Peribunyaviridae*, *Orthobunyavirus*) is an emerging mosquito-borne pathogen endemic to North America [1]*.* The CVV genome is a negative sense, single stranded RNA organized into three distinct segments designated L (large), M (medium) and S (small). The L segment encodes the RNA-dependent RNA polymerase; the M segment encodes two glycoproteins (Gn and Gc) and a non-structural protein (NS_m_); and the S segment encodes the nucleocapsid protein (N) and a second non-structural protein (NSs) [2,3].

CVV is endemic to Canada, Mexico, and the United States, where the virus circulates in mosquitoes and mammals including sheep, cattle and white-tailed deer [4]*.* With prevalence as high as 69% in livestock reported in some regions, CVV is an important cause of embryonic and fetal death or neonatal malformations, resulting in significant economic losses [5–7]. Despite its importance in the livestock industry, no vaccinations or treatments are available. Humans are considered dead-end hosts and while reported human disease is rare, CVV has been associated with neuroinvasive illness [8–11]. Although recent widespread serosurveys are lacking, historic reports suggest seroprevalence may be as high as 40% in some locations in the U.S. [1].

CVV isolates are grouped into 2 lineages [4]. Since 2010 Lineage 2 was shown to have displaced lineage 1 in Connecticut, New York, and Canada [4,12]. Our previous studies demonstrate that *Anopheles* (*An.*) *quadrimaculatus* have increased competence for lineage 2 CVV strains, which likely contributed to the displacement and increased prevalence of CVV in the region over the last decade. In addition, we previously found evidence of segment reassortment in recent strains [12]. For bunyaviruses, reassortments of genome segments during co-infection has played an important role in generating diversity that can confer important alterations to viral fitness and transmissibility in hosts and vectors [13–17].

In order to expand on previous results, we report surveillance testing of CVV from 2017 to 2020 in New York State (NYS). In addition, we determined vector competence of *Aedes* (*Ae.*) *albopictus* for current genetically and phenotypically distinct circulating strains of CVV, including: one lineage 2 strain isolated from a human and two reassortant strains isolated from a human and a pool of mosquitoes. Our results further demonstrate the influence of CVV genetics on transmission and highlight the increasing threat of *Ae. albopictus* as a vector of endemic viruses.

## Materials and methods

### Mosquito surveillance

Adult mosquitoes were collected from 2017 to 2020 in the following regions in NYS: West, Finger Lakes, North, Central, Hudson Valley and Long Island, using CDC light traps baited with CO_2_ [18] or gravid traps [19]. Mosquitoes were identified to species morphologically [20], and females were pooled in groups of 4–50 individuals by trap type, date collected and trap location. Pools were transported on dry ice to the Arbovirus Laboratories, Wadsworth Center, New York State Dept Health, for testing and were stored at −80°C until processed.

Mosquito pools were processed as previously described [21]. Briefly, they were homogenized in 1 ml of mosquito diluent (MD) containing 20% fetal bovine serum, 50 μg of streptomycin per mL, 50 U of penicillin, and 2.5 μg of amphotericin B per mL in phosphate-buffered saline, in a Retsch Mixer Mill set to 24 cycles/s for 2 min. The tubes were then centrifuged for 8 min at 12,000 rpm and the supernatant removed. Approximately 500 μl of supernatant was frozen at −80°C for storage while the remainder was used for RNA extraction [22].

Extraction plates (Thermofisher, Waltham, MA, USA) were prepared on a Tecan Evo 150 liquid handler (Tecan, Morrisvelle, NC, USA) and 50 μL of homogenates were used to extract RNA on a Magmax 96 Express (Applied Biosystems, Waltham, MA, USA) using a MagMax viral isolation kit (Thermofisher, Waltham, MA, USA). A total of 90 μL of homogenized sample RNA was eluted. Real- time RT–PCR assay was performed by using CVV_F (ACAGCCAATGGTGTCGAAAAC), CVV_R (TGCAGGGATGCTAGACAAGATG) primers, and CVV_P (6FAM-CTGACGGTATTGAATCAGCAT-MGBNFQ) probe for CVV detection. Maximum likelihood estimation (MLE) calculations to determine prevalence of CVV in mosquitoes were based upon a program developed by Dr. Brad Biggerstaff as shown on the CDC website, https://www.cdc.gov/westnile/resourcepages/mosqsurvsoft.html [23,24]. Statistical analysis of the virus detection data by year, species, and regions (number of pools) were carried out at a significance level of *p* < 0.05 using OpenEpi, Version 3, open-source calculator–TwobyTwo (https://www.openepi.com/TwobyTwo/TwobyTwo.htm). In addition, we compared the relative mosquitoes abundance between years, regions and the positive mosquito species using a Poisson regression model.

### Genetic analyses of Cache Valley virus

The full genome sequences of two CVV human strains (the lineage 2 Hu-2011 strain isolated from cerebrospinal fluid and the reassortant PA strain isolated from brain tissue) [9,10] and a reassortant isolated from *An. quadrimaculatus* (15041084) obtained from our earlier study were analysed [12]. The two CVV reassortant strains (PA and 15041084) contain lineage 1 L segment and lineage 2 S and M segments and both come from counties in western New York [12]. In addition, PA and 15041084 strains presented different phenotypes in *An. quadrimaculatus* infectivity [12]*.* Nucleotide and amino acid sequence alignments were created with CVV coding regions by using the MegAlign module of the DNAStar software package (https://www.dnastar.com).

### Viral growth kinetics

Confluent monolayers of each cell type in six-well plates were infected in duplicate at a multiplicity of infection (MOI) of 0.01 plaque forming units (pfu) per cell with 100 μl of each virus, and incubated for 1 h at 37°C or 28°C for African Green Monkey kidney (Vero) and *Ae. albopictus* cells (C6/36), respectively, with 5% CO_2_. The inoculum was removed, and cells were washed twice with BA-1 (blood agar media) to remove any remaining virus. Infected plates were then overlaid with 3 ml maintenance media (Eagle minimum essential medium with 2% fetal bovine serum heat inactivated with ½ g/L sodium bicarbonate plus 0.1 mM non-essential amino acids plus 100 U/ml penicillin–streptomycin). Supernatant (100 μl) was sampled at 24 h intervals up to 96 h post-infection (4 days), 20% FBS were added to each sample and stored at −80°C. Titrations were performed in duplicate by plaque assay on Vero cells [25] and mean titres for each time point were calculated. Growth kinetics were compared using repeated measured ANOVA and Tukey’s post hoc tests (GraphPad Prism, Version 5.0) [26].

### Mosquito vector competence

*Ae. albopictus* (Spring Valley, NY, USA; kindly provided by Laura Harrington, Cornell University) were established in 2019 from field-collected eggs. Eggs were hatched in distilled water and maintained under standard rearing conditions (27 ± 1°C; 70% relative humidity; 12:12-h light:dark photoperiod) [25,27]. F7 females were used for the CVV experiments.

Two CVV strains isolated in humans (Hu-2011and PA) and the reassortant 15041084, isolated in mosquitoes [9,10] were used to infect Vero cells at a MOI of 0.01 pfu/cell using 100 μl of each virus and maintained at 37°C, 5% CO_2_. At 48 h following infection, the supernatants were harvested and diluted 1:1 with defibrinated sheep blood plus a final concentration of 2.5% sucrose. In addition, fresh virus supernatants were diluted to 1:100 (Hu-2011) or 1:1000 (15041084) in C6/36 maintenance media before being mixed 1:1 with defibrinated sheep blood with a final concentration of 2.5% sucrose. Only the PA strain supernatants were not diluted before being mixed with defibrinated sheep blood. Female *Ae. albopictus* (5–7 days old) deprived of sugar for 24 h were allowed to feed on the infectious bloods for 45 min via a Hemotek membrane feeding system (Discovery Workshops, Acrington, UK) with a porcine sausage casing membrane at 37°C [28]. Following feeding, females were anesthetized with CO_2_ and fully engorged mosquitoes were transferred to 0.6 L cardboard containers and maintained with 10% sucrose under standard rearing conditions [27] until harvested for testing. A 1 ml aliquot of each blood meal pre-feeding was frozen at −80°C and CVV titres were determined by plaque assay on Vero cells [25]. Three biological replicates were performed for each CVV strain.

Infection, dissemination, and transmission assays were performed on days 7 and 14 post infectious blood meal as previously described [28]. Dissemination rate is the proportion of mosquitoes with infected legs among the infected mosquitoes. Transmission rate is the proportion of mosquitoes with positive saliva among mosquitoes with disseminated infection. Real-time RT–PCR was used to detect CVV following sample processing as previously described [28]. A Fisher’s exact test was used to compare mosquito infection rates, dissemination rates, and transmission rates between groups exposed to distinct CVV isolates at both time points. All statistical analyses were carried out at a significance level of *p* < 0.05 using OpenEpi, Version 3, open source calculator–TwobyTwo (https://www.openepi.com/TwobyTwo/TwobyTwo.htm).

## Results

**Figure 1.**
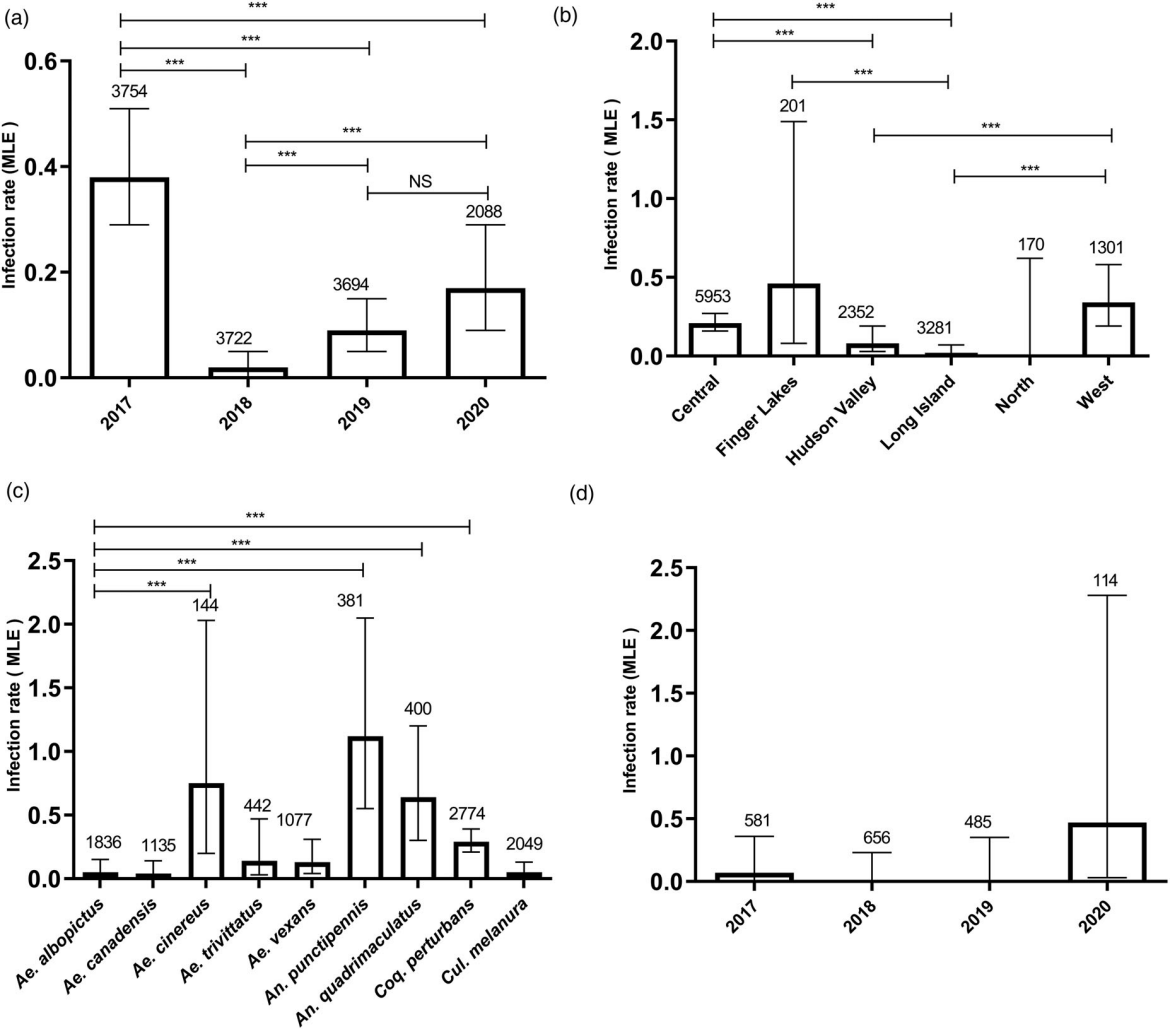
Cache Valley virus prevalence in New York State. Infection rates were calculated by Maximum Likelihood Estimation (MLE) for (A) year (Fisher’s exact test, ***P < 0.01, OR: 5.55, 95% CI: 1.211–51.6), (B) New York State region (Fisher’s exact test, ***P < 0.01, OR: 16.48, 95% CI: 1.186–227.9), (C) mosquito species (Fisher’s exact test, ***P < 0.003, OR: 0.05, 95% CI: 0.004–0.453), or (D) year for *Ae. albopictus*. Bars represent upper and lower limits of infection rate based on 95% confidence levels. Values on top represent number of pools tested. MLEs were calculated using https://www.cdc.gov/westnile/resourcepages/mosqsurvsoft.html. ***P < 0.05.

We tested 441,139 female mosquitoes in 13,258 mosquito pools from 2017 to 2020, yielding a total of 72 CVV positive pools. Comparisons of MLE of prevalence were made by year (Figure 1A), region (Figure 1B), and mosquito species (Figure 1C). CVV activity was detected during each of the 4 years studied with the highest estimates of prevalence in 2017 (0.38, 95% CI [0.29, 0.51]) and 2020 (0.17, 95% CI [0.09, 0.29]) (Figure 1A). Except for 2019 vs 2020, significant differences in prevalence were observed between years studied (Fisher’s exact test, *P* < 0.01, OR: 5.55, 95% CI: 1.211–51.6) (Figure 1A). With the exception of the North region, CVV was detected throughout NYS, with the highest prevalence in the Finger Lakes (0.46, 95% CI [0.08, 1.49]) (Figure 1B). However, Long Island was the only region where the prevalence of CVV pools showed a significant difference compared to Finger Lakes (Fisher’s exact test, *P* < 0.01, OR: 16.48, 95% CI: 1.186–227.9). No significant differences in CVV prevalence was identified for the Central and West regions, nor the Hudson Valley and Long Island regions (Figure 1B). The virus was detected from nine mosquito species, with the highest prevalence in *An. punctipennis* (1.12, 95% CI [0.55, 2.05]), followed by *Ae. cinereus* (0.75, 95% CI [0.2, 2.03]), *An. quadrimaculatus* (0.64, 95% CI [0.3, 1.2]) and *Coquillettidia perturbans*, (0.29, 95% CI [0.21, 0.39]) with no significant differences in CVV prevalence measured between these four species (Figure 1C). Furthermore, the only significant interaction effects were measured when we considered interactions of the relative mosquito abundance between year and region with increase of mosquito abundance in the Finger Lakes in 2019 (*p* = 0.0025), in Long Island in 2019 (*p* = 1.93e−07) and in the West in 2019 (*p* = 0.0155), as compare to Central region in 2017.

*Ae. albopictus* accounted for 9.8% of the total mosquitoes collected (*N* = 43,267, 1836 pools) during the study period (Figure 1D). The invasive mosquito was collected in three different regions including Hudson Valley (11,477 mosquitoes), Long Island (31,779 mosquitoes) and, for the first time, in Northern NYS (11 mosquitoes) in 2020. CVV was detected in 2 pools of *Ae. albopictus* (0.05, 95% CI [0.01, 0.15]) in NYS (Long Island in 2017 and Hudson Valley in 2020) with a similar prevalence as *Ae. canadensis* (0.04, 95% CI [0.01, 0.14]), *Ae. trivittatus* (0.14, 95% CI [0.03, 0.47]), *Ae. vexans* (0.13, 95% CI [0.04, 0.31]) and *Culiseta melanura* (0.05, 95% CI [0.01, 0.13]) (Figure 1C). This represents the first isolation of CVV from *Ae. albopictus* in NYS.

Sequence analysis of the two reassortant strains (PA and 15041084) revealed 0.3%, 0.4%, 0.3% nucleotide divergence and 1, 10, and 6 amino acid differences for the S, M, and L segments, respectively. In addition, the PA and Hu-2011 (Lineage 2) strains showed 0.5%, 0.7% and 6.3% nucleotide divergence, and 1, 12, and 29 amino acid difference for the S, M, and L segments, respectively (Table 1). For the S segment, the only unique amino acid, F70S, occurred in the PA strain NSs protein. For the M segment, the NSm protein of 15041084 and Hu-2011 strains are identical and differed from the PA strain by two amino acids, V371G and K450Q. The Gc protein of the PA strain was found to be more divergent, with 8 and 10 amino acid differences relative to the 15041084 and Hu-2011strains, respectively. With the exception of amino acid changes T666A and V845D, the Gc protein of the 15041084 and Hu-2011 strains were identical (Table 1). Consistent with other lineage 2 strains, the L protein of the Hu-2011strain was most distinct from the reassortant strains, with 27 and 29 amino acid differences in comparison with the 15041084 and PA strains, respectively (Table 1).

**Table 1.** Amino acid differences among Cache Valley virus (CVV) strains used for vector competence and growth kinetic studies.

			CVV strains, amino acids		
Segment	Amino acid position	Protein	PA	15041084	Hu-2011
S	70	NSs	S	F	F
M	371	NSm	V	G	G
	450	NSm	K	Q	Q
	488	Gc	I	M	M
	521	Gc	I	V	V
	589	Gc	I	T	T
	603	Gc	Q	R	R
	609	Gc	A	T	T
	627	Gc	S	N	N
	666	Gc	T	T	A
	683	Gc	K	N	N
	697	Gc	I	T	T
	845	Gc	V	V	I
L	6	L	H	H	Y
	59	L	I	I	V
	91	L	M	M	I
	196	L	D	D	G
	223	L	D	D	N
	242	L	T	T	A
	243	L	T	A	A
	295	L	I	I	T
	319	L	G	G	S
	345	L	K	K	R
	365	L	L	L	M
	408	L	A	G	G
	736	L	K	K	R
	790	L	G	G	E
	863	L	K	K	R
	905	L	K	K	R
	1302	L	N	N	D
	1360	L	H	H	Q
	1362	L	G	G	N
	1436	L	S	S	D
	1503	L	G	S	S
	1632	L	T	T	N
	1700	L	I	I	V
	1708	L	N	D	N
	1850	L	V	F	V
	1889	L	F	F	Y
	1900	L	N	N	S
	1912	L	I	I	M
	1932	L	T	T	A
	1948	L	I	V	V
	2043	L	S	S	N
	2190	L	H	H	C

**Figure 2.**
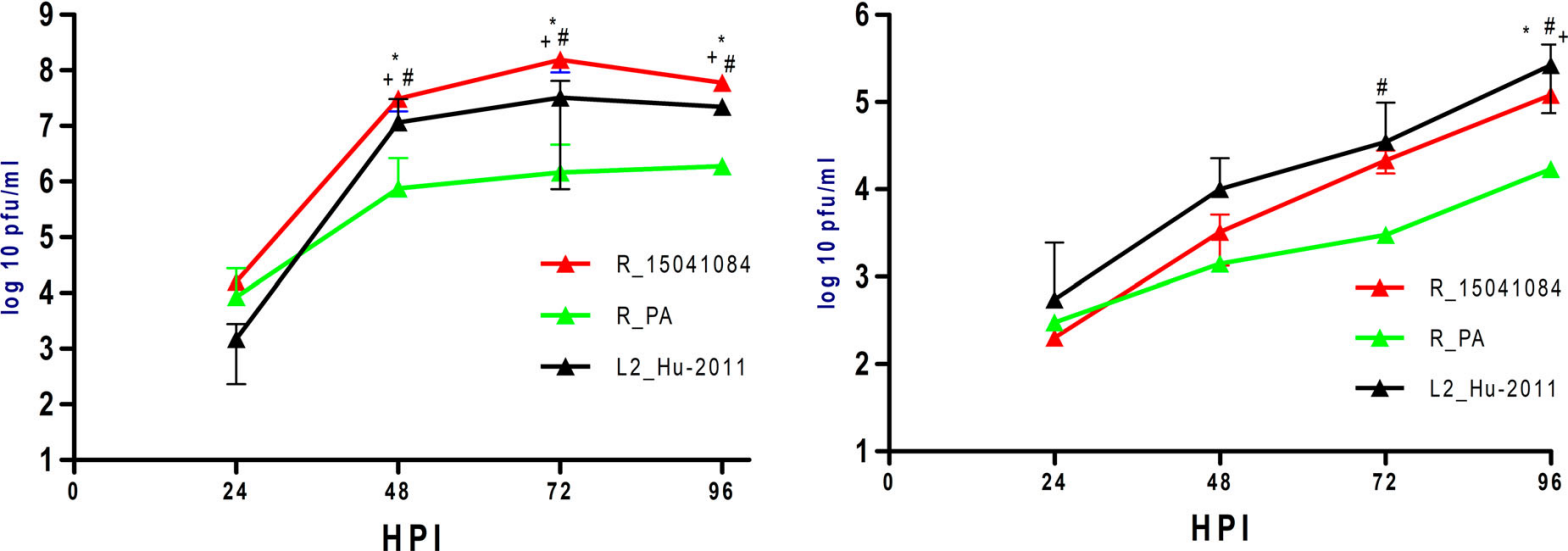
Growth kinetics of unique Cache Valley virus strains in cell culture. Kinetics were determined in (A) mammalian (Vero) or (B) mosquito (C6/36) cells. Points represent means of duplicate values ± standard deviation. ^#^Denotes significant difference (Two-way ANOVA, *p* < 0.001 by Tukey post hoc test) between PA and Hu-2011 CVV strains at indicated time points in both cell lines. *Denotes significant difference (Two-way ANOVA, *p* < 0.001 by Tukey post hoc test) between Hu-2011 and 15041084 CVV strains at indicated time points in both cell lines. ^+^Denotes significant difference (Two-way ANOVA, *p* < 0.001 by Tukey post hoc test) between PA and 15041084 CVV strains at indicated time points in both cell lines. A significant effect independent of the time was measured in both cell lines (Two-way ANOVA, *p* < 0.001).

Growth kinetics of PA, 15041084 and Hu-2011 were compared on Vero (37°C) and C6/36 cells (28°C) (Figure 2). The 15041084 strain replicated to significantly higher titres than the two other CVV strains at 48, 72 and 96 h on Vero cells (*t*-test, *p* < 0.05) with ∼8 log_10_ PFU/mL peak viral titre observed at 72 h (Figure 2A). With the exception of 24 h, significant differences were also identified between Hu-2011 (∼7 log_10_ PFU/mL peak viral titre at 72 h) and PA (∼6 log_10_ PFU/mL peak viral titre at 96 h) strains at all other time points on Vero cells (*t*-test, *p* < 0.05; Figure 2A). In C6/36 cells, no significant differences were observed at 24 and 48 h between strains. However, the PA strain showed significantly lower titres as compared to 15041084 at 96 h, and Hu-2011 at 72 and 96 h (*t*-test, *p* < 0.05; Figure 2B).

Vector competence assays with *Ae. albopictus* for CVV Hu-2011, PA, and 15041084 were conducted to determine the transmission potential of *Ae. albopictus* for genetically distinct CVV strains (Table 2). When mosquitoes fed on high virus titres (≥5.5 log_10_PFU/mL), infection rates were 100.0% for all three CVV isolates at 14 days post-infection (dpi). Dissemination rates for both Hu-2011 and 15041084 were also 100% yet, strikingly, mosquitoes exposed to CVV PA did not show evidence of dissemination. Transmission rates for CVV Hu-2011 and CVV 15041084 were also high, 61.9% and 48.0%, respectively (Table 2). Significant reduction in infection rates were observed following exposure to blood meals with lower input titres for CVV 15041084 (4.2 to 3.6 log_10_ PFU/mL) (Fisher’s exact test, *P* < 0.001, OR: 32.5, 95% CI: 6.675–175.4; *P* < 0.001, OR: 6.538, 95% CI: 1.739-27.14) and the lineage 2 CVV Hu-2011 (5 to 4.5 log_10_ PFU/mL; Fisher’s exact test, *P* < 0.001, OR: 37.92, 95% CI: 4.671–1638; *P* < 0.001, OR: 43.5, 95% CI: 5.322–1872) at 7 and 14 dpi, respectively. However, no significant differences in the dissemination rates were identified for CVV 15041084 at 7 and 14 dpi. In addition, exposure to lower blood meal titres significantly reduced CVV Hu-2011 dissemination rate at 7 dpi (Fisher’s exact test, *P* < 0.02, OR: 12.89, 95% CI: 1.023–655.8) and transmission rates at 14 dpi (Fisher’s exact test, *P* < 0.009, OR: 8.182, 95% CI: 1.304–85.84). Furthermore, between 7 and 14 dpi significant increases in transmission rates were observed for the CVV 15041084 (*P* < 0.04, OR: 0.2424, 95% CI: 0.03745–1.209) and CVV Hu-2011 (*P* < 0.001, OR: 0.1528, 95% CI: 0.03948-0.5573). Despite this, transmission was measured for both strains at the lowest doses utilized (Table 2). Our results indicate that *Ae. albopictus* is a competent vector for CVV Hu-2011 and the CVV 15041084, but not for the human reassortant (PA) isolate, which did not disseminate from the mosquito midgut (Table 2).

**Table 2.** Infection, dissemination and transmission rates of Ae. albopictus following exposure to distinct Cache Valley virus (CVV) strains.

Experiment	CVV strains	Blood meal titrelog_10_ PFU/ml	7 Dpi			14 Dpi		
			Infectionno (%)	Disseminationno (%)	Transmissionno (%)	Infectionno (%)	Disseminationno (%)	Transmissionno (%)
1	R_15041084	7.4	Nt	Nt	Nt	43/43 (100)	43/43 (100)	21/43 (48.0)
	L2_Hu2011	6.7	Nt	Nt	Nt	42/42 (100)	42/42 (100)	26/42 (61.9)
	R_PA	5.5	Nt	Nt	Nt	41/41 (100)	0/42 (0)	Nt
2	R_15041084	4.2	26/30 (86.7)	25/26 (96.2)	3/25 (12)	25/30 (83.33)	25/25 (100.0)	9/25 (36)^ˠ^
	L2_Hu2011	5.0	30/30 (100.0)	30/30 (100.0)	6/30 (20.0)	30/30 (100)	29/30 (96.67)	18/29 (62.1)^ˠ^
	R_PA	5.2	21/30 (70)	0/21 (0.0)	Nt	23/30 (73.6)	0/23 (0.0)	Nt
3	R_15041084	3.6	5/30 (16.7)*	4/5 (80.0)	2/4 (50.0)	13/30 (43.33)*	11/13 (84.6)	3/11 (27.3)
	L2_Hu2011	4.5	13/30 (43.3)*	9/13 (69.2)^#^	1/9 (11.1)	12/30 (40.0) *	12/12 (100.0)	2/12 (16.7)^+^
	R_PA	5.3	7/30 (23.3)	0/7 (0.0)	Nt	20/30 (66.67)	0/20 (0.0)	Nt

Nt: not tested.

*P < 0.05: comparaison of mosquito infection rates after feeding on the same virus strains with different blood meal titres.

^#^ *P* < 0.05: comparaison of mosquito dissemination rates after feeding on the same virus strains with different blood meal titres.

^+^ *P* < 0.05: comparaison of mosquito transmission rates after feeding on the same virus strains with different blood meal titres.

^ˠ^ *P* < 0.05: comparaison of mosquito transmission rates at different time points for the same strain and experiment.

## Discussion

Our study confirmed yearly variability in CVV activity in NYS and association of the virus with various mosquito genera including *Aedes, Anopheles, Coquillettidia* and *Culiseta,* as described previously [12,29]. In addition, our results corroborate previous findings showing higher prevalence of CVV in *An. punctipennis* and *An. quadrimaculatus* [12,29]. Notably, CVV was detected for the first time in *Ae. albopictus* collected in NYS, suggesting the potential involvement of this species in the transmission cycle. Previous isolations of CVV from *Ae. albopictus* have been reported in Connecticut and New Jersey [4,30]. Interestingly, all CVV isolates from *Ae. albopictus* in northeastern USA belong to lineage 2 [31].

The Asian tiger mosquito (*Ae. albopictus*) is a highly invasive species that has been introduced into the U.S. and has become permanently established in at least 27 states, including NYS [32]. Our retrospective surveillance data in NYS from 2000 to 2016 confirmed that *Ae. albopictus* populations are well established in the Long Island and Hudson Valley regions [12,33]; however, the first detection in the northern NYS suggests that this species may be continuing to expand its range in the northeastern U.S. The distribution and spread of this species is coincidental with the high prevalence of CVV in white-tailed deer in NYS [34]. However, many factors, including mosquito, host feeding preference (mammalian) and viral evolution, could be contributing to the increase of CVV circulation in the region.

Previous studies have shown that both the M and L segments of bunyaviruses have a role in determining host virulence and neuropathogenicity, as well as mosquito infectivity [35–39]. We previously demonstrated increased infectivity of lineage 2 CVV strains in *Anopheles*, consistent with increased prevalence following lineage 1 displacement in the Northeast [12,29]. In addition, the three CVV reassortant mosquito isolates that contained only the lineage 1 L segment were more infectious than other lineage 1 strains, suggesting a role for the S and/or M segments in the increased mosquito infectivity of lineage 2 strains [12]. However, the human reassortant strain (PA) sharing the lineage 1 L segment and the lineage 2 S and M segments with the mosquito reassortant strains was not transmitted by *An. quadrimaculatus* [12]. The high susceptibility of *Ae. albopictus* to CVV obtained in our results are similar to the reports of others [31,40]. In this study, using *Ae. albopictus* mosquitoes, we confirmed the PA phenotype observed previously with *An. quadrimaculatus.* We additionally demonstrated that this strain was attenuated in vertebrate and invertebrate cell culture. Together, our data suggest that individual mutations, in addition to segment reassortment, can play a critical role in determining CVV fitness.

Our sequence analysis revealed amino acid difference in NSs (1aa), NSm (2 aa), Gc (8 aa) and L (6 aa) proteins between the two reassortant strains (PA and 15041084). Previous studies showed that NSs and NSm proteins are not essential for bunyaviruses viability but deletions of the NSs proteins reduced viral replication [41–43]. Furthermore, the NSs protein is a major virulence factor and plays an important role in viral evasion of innate immunity [41,42]. Rift Valley Fever virus (RVFV) NSs has been shown to influence dissemination rates in *Ae. aegypti* while deletion of the RVFV NSm was sufficient to nearly abolish mosquito infection [44,45]. Moreover, for orthobunyaviruses it is generally accepted that both glycoproteins are required for virus entry. Although little is known about individual Gn and Gc protein functions, it is suggested that Gc is the attachment protein for mammalian and mosquito cells [46–50]. For La Crosse virus and California encephalitis virus, the specificity of virus-vector interactions is thought to be strongly influenced by the efficiency of the fusion function of the Gc (G1) envelope glycoprotein operating at the midgut level in the arthropod vector [46,50]. Understanding of the biological function of the non-structural and Gc proteins of CVV is critical to uncovering the role of individual mutations in host-specific fitness and transmissibility of emergent CVV strains.

The invasiveness of *Ae. albopictus*, as well his zoophilic behaviour with a preference for human blood [32] and its potential to transmit endemic and invasive arboviruses, could increase the threat from local and introduced viruses in the Northeast U.S. Further, the general increase in CVV activity, the capacity for CVV transmission, and the influence of viral genetics on vector competence, suggest *Ae. albopictus* are likely to contribute to the expanding threat of CVV transmission and disease.

## References

[1] Waddell L, Pachal N, Mascarenhas M, et al. Cache Valley virus: a scoping review of the global evidence. Zoonoses Public Heal. 2019;66:739–758. 10.1111/zph.12621 PMC6851749 31254324

[2] Dunlop JI, Szemiel AM, Navarro A, et al. Development of reverse genetics systems and investigation of host response antagonism and reassortment potential for Cache Valley and Kairi viruses, two emerging orthobunyaviruses of the Americas. PLoS Negl Trop Dis. 2018;12:e0006884. 30372452 10.1371/journal.pntd.0006884 PMC6245839

[3] Elliott RM. Molecular biology of the Bunyaviridae. J Gen Virol. 1990;71:501–522. 2179464 10.1099/0022-1317-71-3-501

[4] Armstrong PM, Andreadis TG, Anderson JF. Emergence of a new lineage of Cache Valley virus (Bunyaviridae : Orthobunyavirus) in the Northeastern United States. Am J Trop Med Hyg. 2015;93:11–17. 25962774 10.4269/ajtmh.15-0132 PMC4497881

[5] Chung SI, Livingston CWJ, Jones CW, et al. Cache Valley virus infection in Texas sheep flocks. J Am Vet Med Assoc United States. 1991;199:337–340. 1917638

[6] Meyers MT, Bahnson CS, Hanlon M, et al. Management factors associated with operation-level prevalence of antibodies to Cache Valley virus and other Bunyamwera serogroup viruses in sheep in the United States. Vector-Borne Zoonotic Dis. 2015;15:683–693. 26565774 10.1089/vbz.2015.1810 PMC12407189

[7] Uehlinger FD, Wilkins W, Godson DL, et al. Seroprevalence of Cache Valley virus and related viruses in sheep and other livestock from Saskatchewan, Canada. Can Vet J. 2018;59:413–418. 29606729 PMC5855288

[8] Campbell GL, Mataczynski JD, Reisdorf ES, et al. Second human case of Cache Valley virus disease. Emerg Infect Dis. 2006;12:854–856. 16704854 10.3201/eid1205.051625 PMC3374447

[9] Nguyen NL, Zhao G, Hull R, et al. Cache Valley virus in a patient diagnosed with aseptic meningitis. J Clin Microbiol. 2013;51:1966–1969. 23515536 10.1128/JCM.00252-13 PMC3716113

[10] Yang Y, Qiu J, Snyder-Keller A, et al. Fatal Cache Valley virus meningoencephalitis associated with rituximab maintenance therapy. Am J Hematol. 2018;93:590–594. 29282755 10.1002/ajh.25024 PMC6037180

[11] Wilson MR, Suan D, Duggins A, et al. A novel cause of chronic viral meningoencephalitis: Cache Valley virus. Ann Neurrology. 2017;82:105–114. 10.1002/ana.24982 PMC5546801 28628941

[12] Dieme C, Ngo KA, Tyler S, et al. Role of Anopheles mosquitoes in Cache Valley virus lineage displacement, New York, USA. Emerg Infect Dis. 2022;28:303–313. 35075998 10.3201/eid2802.203810 PMC8798675

[13] Horne KME, Vanlandingham DL. Bunyavirus-vector interactions. Viruses. 2014;6:4373–4397. 25402172 10.3390/v6114373 PMC4246228

[14] Elliott RM. Bunyaviruses and climate change. Clin Microbiol Infect European Soc Clinic Microbiol Infect Dis. 2009;15:510–517. 10.1111/j.1469-0691.2009.02849.x 19604275

[15] Pringle CR. Genetics and genome segment reassortment. In: Elliott RM, editor. The Bunyaviridae. The Viruses. Boston (MA): Springer; 1996. DOI:10.1007/978-1-4899-1364-7_8

[16] Mcdonald SM, Nelson MI, Turner PE, et al. Reassortment in segmented RNA viruses: mechanisms and outcomes. Nat Rev Microbiol. 2016;14:448–460. 27211789 10.1038/nrmicro.2016.46 PMC5119462

[17] Vijaykrishna D, Mukerji R, Smith GJD. RNA virus reassortment: an evolutionary mechanism for host jumps and immune evasion. PLoS Pathog. 2015;11:1–6. 10.1371/journal.ppat.1004902 PMC4497687 26158697

[18] Newhouse VF, Siverly RE. St. Louis encephalitis virus from mosquitoes in southwestern Indiana, 1964. J Med Entomol. England. 1966;3:340–342. 10.1093/jmedent/3.3-4.340 5986756

[19] Reiter P. A portable battery-powered trap for collecting gravid Culex mosquitoes. Mosq News. 1983;43:496–498.

[20] Means RG. Mosquitoes of New York, part II: genera of culicidae other than Aedes occuring in New York. New York State Museum. 1987;430B:1–180.

[21] Oliver J, Lukacik G, Kokas J, et al. Twenty years of surveillance for Eastern equine encephalitis virus in mosquitoes in New York State from 1993 to 2012. Parasit Vecs. 2018;11:362. 10.1186/s13071-018-2950-1 PMC6019270 29941031

[22] Kauffman EB, Jones SA, Dupuis AP, et al. Virus detection protocols for West Nile virus in vertebrate and mosquito specimens. J Clin Microbiol. 2003;41:3661–3667. 12904372 10.1128/JCM.41.8.3661-3667.2003 PMC179779

[23] Bialosuknia SM, Tan Y, Zink SD, et al. Evolutionary dynamics and molecular epidemiology of west Nile virus in New York state: 1999–2015. Virus Evol. 2019;5:1–13. 10.1093/ve/vez020 PMC6642743 31341640

[24] Eastwood G, Shepard JJ, Misencik MJ, et al. Local persistence of novel regional variants of La Crosse virus in the Northeast USA. Parasit Vec BioMed Central. 2020;13:1–8. 10.1186/s13071-020-04440-4 PMC7659055 33176861

[25] Dieme C, Ciota AT, Kramer LD. Transmission potential of Mayaro virus by Aedes albopictus, and Anopheles quadrimaculatus from the USA. Parasit Vec BioMed Central. 2020;13:4–9. 10.1186/s13071-020-04478-4 PMC7724717 33298165

[26] Kuo L, Jaeger AS, Banker EM, et al. Reversion to ancestral Zika virus NS1 residues increases competence of Aedes albopictus. PLoS Pathog. 2020;16:1–16. 10.1371/journal.ppat.1008951 PMC7588074 33052957

[27] Ciota AT, Matacchiero AC, Kilpatrick AM, et al. The effect of temperature on life history traits of Culex mosquitoes. J Med Entomol. 2014;51:55–62. 24605453 10.1603/me13003 PMC3955846

[28] Ciota AT, Bialosuknia SM, Zink SD, et al. Effects of Zika virus strain and Aedes mosquito species on vector competence. Emerg Infect Dis. 2017;23:1110–1117. 28430564 10.3201/eid2307.161633 PMC5512477

[29] Andreadis TG, Armstrong PM, Anderson JF, et al. Spatial-temporal analysis of Cache Valley virus (Bunyaviridae: Orthobunyavirus) infection in anopheline and culicine mosquitoes (Diptera: Culicidae) in the Northeastern United States, 1997–2012. Vector-Borne Zoonotic Dis. 2014;14:763–773. 25325321 10.1089/vbz.2014.1669 PMC4208611

[30] Armstrong PM, Anderson JF, Farajollahi A, et al. Isolations of Cache Valley virus from Aedes albopictus (Diptera : Culicidae) in New Jersey and evaluation of its role as a regional arbovirus vector. J Med Entomol. 2013;50:1310–1314. 24843937 10.1603/me13099

[31] Ayers VB, Huang YJS, Lyons AC, et al. Infection and transmission of Cache Valley virus by Aedes albopictus and Aedes aegypti mosquitoes. Parasit Vectors. 2019;12:384. 31366369 10.1186/s13071-019-3643-0 PMC6670168

[32] Kraemer MUG, Reiner RC, Brady OJ, et al. Past and future spread of the arbovirus vectors Aedes aegypti and Aedes albopictus. Nat Microbiol. 2019;4:854–863. 30833735 10.1038/s41564-019-0376-y PMC6522366

[33] Shragai T, Harrington LC. Aedes albopictus (Diptera: Culicidae) on an invasive edge: abundance, spatial distribution, and habitat usage of larvae and pupae across urban and socioeconomic environmental gradients. J Med Entomol. 2019;56:472–482. 30566612 10.1093/jme/tjy209

[34] Dupuis AP, Prusinski MA, Russell A, et al. Serologic survey of mosquito-borne viruses in hunter-harvested white-tailed deer (odocoileus virginianus), New York State. Am J Trop Med Hyg. 2020;104:593–603. 33350367 10.4269/ajtmh.20-1090 PMC7866319

[35] Hughes HR, Lanciotti RS, Blair CD, et al. Full genomic characterization of California serogroup viruses, genus Orthobunyavirus, family Peribunyaviridae including phylogenetic relationships. Virology. 2017;512:201–210. 28985574 10.1016/j.virol.2017.09.022

[36] Endres MJ, Griot C, Gonzalez-Scarano F, et al. Neuroattenuation of an avirulent bunyavirus variant maps to the L RNA segment. J Virol. 1991;65:5465–5470. 1895395 10.1128/jvi.65.10.5465-5470.1991 PMC249038

[37] Briese T, Calisher CH, Higgs S. Viruses of the family Bunyaviridae: are all available isolates reassortants? Virology. 2013;446:207–216. 24074583 10.1016/j.virol.2013.07.030

[38] Cheng LL, Rodas JD, Schultz KT, et al. Potential for evolution of California serogroup bunyaviruses by genome reassortment in Aedes albopictus. Am J Trop Med Hyg. 1999;60:430–438. 10466972 10.4269/ajtmh.1999.60.430

[39] Fausta Dutuze M, Nzayirambaho M, Mores CN, et al. A review of Bunyamwera, Batai, and Ngari viruses: understudied Orthobunyaviruses with potential one health implications. Front Vet Sci. 2018;5:1–9. 29707545 10.3389/fvets.2018.00069 PMC5906542

[40] Chan KK, Auguste AJ, Brewster CC, et al. Vector competence of Virginia mosquitoes for Zika and Cache Valley viruses. Parasit Vec BioMed Central. 2020;13:1–9. 10.1186/s13071-020-04042-0 PMC7147054 32276649

[41] Eifan S, Schnettler E, Dietrich I, et al. Non-structural proteins of arthropod-borne bunyaviruses: roles and functions. Viruses. 2013;5:2447–2468. 24100888 10.3390/v5102447 PMC3814597

[42] Elliott RM. Orthobunyaviruses: recent genetic and structural insights. Nat Rev Microbiol. 2014;12:673–685. 25198140 10.1038/nrmicro3332

[43] Szemiel AM, Failloux AB, Elliott RM. Role of Bunyamwera Orthobunyavirus NSs protein in infection of mosquito cells. PLoS Negl Trop Dis. 2012;6:e1823. 23029584 10.1371/journal.pntd.0001823 PMC3459826

[44] Kading RC, Crabtree MB, Bird BH, et al. Deletion of the NSm virulence gene of Rift Valley Fever virus inhibits virus replication in and dissemination from the midgut of Aedes aegypti mosquitoes. PLoS Negl Trop Dis. 2014;8:17–18. 10.1371/journal.pntd.0002670 PMC3923680 24551252

[45] Won S, Ikegami T, Peters CJ, et al. NSm protein of Rift Valley Fever virus suppresses virus-induced apoptosis. J Virol. 2007;81:13335–13345. 17913816 10.1128/JVI.01238-07 PMC2168885

[46] Sundin DR, Beaty BJ, Nathanson N, et al. A G1 glycoprotein epitope of La Crosse virus: a determinant of infection of Aedes triseriatus. Science. 1987;80:235. 10.1126/science.3810159 3810159

[47] Plassmeyer ML, Soldan SS, Stachelek KM, et al. California serogroup Gc (G1) glycoprotein is the principal determinant of pH-dependent cell fusion and entry. Virology. 2005;338:121–132. 15923017 10.1016/j.virol.2005.04.026

[48] Pekosz A, Griot C, Nathanson N, et al. Tropism of bunyaviruses: evidence for a G1 glycoprotein-mediated entry pathway common to the California serogroup. Virology. 1995;214:339–348. 8553534 10.1006/viro.1995.0043

[49] Hacker JK, Hardy JL. Adsorptive endocytosis of california encephalitis virus into mosquito and mammalian cells: A role for G1. Virology. 1997;235:40–47. 9300035 10.1006/viro.1997.8675

[50] Hacker JK, Volkman LE, Hardy JL. Requirement for the G1 protein of California encephalitis virus in infection in vitro and in vivo. Virology. 1995;206:945–953. 7531919 10.1006/viro.1995.1017

